# Editorial: Prevention, assessment and treatment of clinical issues related to endurance exercise and sports

**DOI:** 10.3389/fphys.2022.1082237

**Published:** 2023-01-04

**Authors:** Daniel Rojas-Valverde, Martin Burtscher, Gregoire P. Millet, Volker Scheer, Pantelis T. Nokolaidis, Beat Knechtle

**Affiliations:** ^1^ Sport Injury Clinic (Rehab&Readapt), Escuela Ciencias del Movimiento Humano y Calidad de Vida (CIEMHCAVI), Universidad Nacional, Heredia, Costa Rica; ^2^ Núcleo de Estudios para el Alto Rendimiento y la Salud (CIDISAD-NARS), Escuela Ciencias del Movimiento Humano y Calidad de Vida (CIEMHCAVI), Universidad Nacional, Heredia, Costa Rica; ^3^ Department of Sport Science, University of Innsbruck, Innsbruck, Austria; ^4^ Institute of Sport Sciences, University of Lausanne, Lausanne, Switzerland; ^5^ Ultra Sports Science Foundation, Pierre-Benite, France; ^6^ School of Health and Caring Sciences, University of West Attica Athens, Athens, Greece; ^7^ Institute of Primary Care, University of Zurich, Zurich, Switzerland

**Keywords:** health, running, cycling, cardiovascular, renal, issues. illness

Despite being particularly demanding owing to various internal, external, and even situational variables, endurance exercise and sports have shown to be an ally for the health of many people of multiple ages and biopsychosocial situations. However, in recent years, several clinical disorders connected to inappropriate endurance sport practice have been reported, coinciding with a rise in the popularity of such sports (e.g., cycling, cross-country skiing, running, kayaking, rowing, swimming, triathlon, and hiking) (Moseid et al., 2018; Mann et al., 2021). These clinical concerns can result in physical and physiological changes and have significant health consequences in the short and long term.

Some conditions associated with endurance training and competition include neuromuscular, tendon and bone injuries, dehydration, acute kidney injury, gastrointestinal problems, rhabdomyolysis, epithelial injuries, cardiovascular strain and injuries, liver function changes, and exercise-associated collapse (Scheer et al., 2021).

As a result, it is critical to prevent, identify, monitor, and treat certain health issues to avoid future difficulties. There are now some methods, approaches, and procedures available to help athletes prevent and treat acute and chronic illnesses, allowing stakeholders to preserve athletes’ health and performance (Hoffman et al., 2014). Despite advances in understanding, there are always unanswered questions that require further research and data to be decisive. As a result, more in-depth analysis and scientific evidence are needed that allow for the development of new insights that aid in the clarification of the best techniques for preventing, assessing, and treating clinical issues that eventually cause changes in the functional capacity and performance of endurance practitioners.

As in other populations, there are contextual, family and sociocultural problems that could aggravate or exacerbate the potential deterioration in acute health of active people. In this sense, Morrison et al. demonstrated improved cardiorespiratory fitness (CRF) in Slovenian schoolchildren (6–14 years) following the implementation of population-wide physical fitness strategies (Morrison et al.). Those strategies, e.g., new physical education curricula and national physical activity programs, may have considerably contributed to the reversal of negative trends in CRF, i.e., the 20-m shuttle run (20mSRT) observed at the turn of the millennium. The subsequent increase (8.2%) in the 20mSRT performance (and associated improvement of the cardiovascular health risk) was observed across all age groups of boys and girls (Morrison et al.).

Concerning monitoring physical capacities, as has been shown in the study by Morrison et al., it is necessary to explore new options for measuring the sports and health conditions of endurance athletes to control potential risks. Considering the abovementioned, the study by Walsh et al. evaluated the reliability of a protocol measuring peak eccentric (ECC) torque generated by the lower limb during semi-recumbent ECC cycling (Walsh et al.). The authors showed that the protocol applied is appropriate for the reliable determination of peak ECC torque, which may be useful for prescribing workloads for semi-recumbent ECC cycling (Walsh et al.).

Specifically, in endurance athletes, there are different functions and structures affected by exposure to high volumes of exercise, one of the systems recently studied is the gastrointestinal system. Based on a case series study and the implementation of a gastrointestinal assessment protocol during exercise (GastroAxEx), Gaskell et al. suggest that gastrointestinal symptoms (GIS) during practice in endurance athletes are rather associated with gastrointestinal functioning and feeding tolerance than gastrointestinal integrity and systemic aspects (Gaskell et al.). Thus, GastroAxEx may represent a valuable individualised therapeutic intervention approach.

One of the most recently studied problems associated with endurance exercise is acute kidney injury, especially in ultra-endurance runners. Atkins et al. investigated the effects of running the Boston Marathon on biomarkers of acute kidney injury (AKI) (Atkins et al.). Besides sex differences, the authors found that renal stress biomarkers remained elevated 24-h post-marathon and that some participants even failed to rehydrate within this period (Atkins et al.). These conclusions contrast with data reported by previous studies in other marathons (Clarkson, 2007; Rojas-Valverde et al., 2020), these differences in the results may be due to sampling particularities, or technical variances in the evaluation.

Although these effects have been presented and studied in endurance athletes, more recently, cases of acute kidney injury have been reported in other sports, such as football. In this sense, deeper monitoring of the relationship between load and kidney damage in these sports is necessary. Wołyniec et al. evaluated the effects of free sugars and the sweetener xylitol on kidney function and potential kidney injury post-exercise in semi-professional football players (Wołyniec et al.). Although biomarkers of AKI were present in all experiments, they were most pronounced after xylitol intake. Moreover, side effects, e.g., diarrhoea, were also most prevalent after xylitol (Wołyniec et al.). Thus, sweeteners seem not to be an appropriate alternative to sugars.

Endurance sports practice is becoming increasingly popular among adolescents, masters, and female athletes (Scheer et al., 2021). Participation has several health advantages, but endurance sports can seldom cause major adverse effects. However, a growing body of research suggests endurance sports practice may have long-term health effects, notably for the cardiovascular, pulmonary, and musculoskeletal systems. More research is needed to determine whether this applies to sensitive individuals and whether it affects large training volumes or the periodic stress of racing. Future research is required better to understand illness prevalence and pathogenesis in endurance athletes.

There may be particular interest in focusing future research on clarifying the physiological pathway of some acute and chronic responses to endurance exercise, epidemiological evidence in clinical endurance issues, exploration of how contextual and situational factors lead to clinical matters, analyse of how genetics influence acute and chronic problems in endurance athletes, redirect nutritional approaches to prevent and treat clinical problems, explore new assessment methods, means and strategies for the identification of clinical issues.

Also, new tools, technologies, and protocols must be studied to assess, monitor, and counteract the adverse effects of participating in endurance sports. This Research Topic represents a call to the scientific community to pay attention to possible health problems in the short and long term of the practice of endurance exercise and sports; we hope that facilitating this scientific dissemination platform will be the prelude to delving into this matter.
